# Supplementary material for: Chemical Synthesis, Pharmacokinetic Properties and Biological Effects of JM-00266, a Putative Non-Brain Penetrant Cannabinoid Receptor 1 Inverse Agonist
Source: Int J Mol Sci. 2022 Mar 8;23(6):2923. doi: 10.3390/ijms23062923 (PMC8949893; doi:10.3390/ijms23062923)
Supplement: Supplementary file 1 [file ijms-23-02923-s001.zip › ijms-1592514-supplementary.pdf]

International Journal of
Molecular Sciences

MDPI

Article

Chemical Synthesis, Pharmacokinetic Properties and Biological Effects of JM-00266, a Putative Non-Brain Penetrant Cannabinoid Receptor 1 Inverse Agonist

Tania Muller [1], Laurent Demizieux [1], Stéphanie Troy-Fioramonti [1], Chloé Buch [1], Julia Leemput [1], Christine Belloir [2], Jean-Paul Pais de Barros [3], Tony Jourdan [1], Patricia Passilly-Degrace [1], Xavier Fioramonti [2,4], Anne-Marie Le Bon [2], Bruno Vergès [1], Jean-Michel Robert [5] and Pascal Degrace [1,*]

[1] Equipe Physiopathologie des dyslipidémies, Unité Mixte de Recherche Université de Bourgogne Franche-Comté - Institut National de la Santé et de la Recherche Médicale (UMR-INSERM) 1231, 21000 Dijon, France; tania.muller67@gmail.com (T.M.); laurent.demizieux@u-bourgogne.fr (L.D.); stephanie.fioramonti@hotmail.fr (S.T.-F.); chloejeanne.buch@gmail.com (C.B.); julia.leemput@u-bourgogne.fr (J.L.); tony.jourdan@u-bourgogne.fr (T.J.); patricia.degrace@u-bourgogne.fr (P.P.-D.); bruno.verges@u-bourgogne.fr (B.V.)

[2] Centre des Sciences du Goût et de l'Alimentation (CSGA), AgroSup Dijon, Centre National de la Recherche Scientifique (CNRS), Institut National de Recherche pour l'Agriculture, l'Alimentation et l'Environnement (INRAE), Université de Bourgogne Franche-Comté, F-21000 Dijon, France; christine.belloir@inrae.fr (C.B.); xavier.fioramonti@inrae.fr (X.F.); anne-marie.le-bon@inrae.fr (A.-M.L.B.)

[3] Plateforme de Lipidomique, Unité Mixte de Recherche Université de Bourgogne Franche-Comté - Institut National de la Santé et de la Recherche Médicale (UMR-INSERM) 1231, 21000 Dijon, France; jppais@u-bourgogne.fr

[4] Unité de Nutrition et Neurobiologie Intégrative (NutriNeuro), Unité Mixte de Recherche Université de Bordeaux - Institut National de Recherche pour l'Agriculture, l'Alimentation et l'Environnement (UMR-INRAE) 1286, F-33000 Bordeaux, France

[5] Unité de Recherche Cibles et Médicaments des Infections et de l'Immunité (UR115 IICiMed), Institut de Recherche en Santé 2 Nantes Université, F-44200 Nantes, France; jean-michel.robert@univ-nantes.fr

* Correspondence: pascal.degrace@u-bourgogne.fr

Citation: Muller, T.; Demizieux, L.; Troy-Fioramonti, S.; Buch, C.; Leemput, J.; Belloir, C.; Pais de Barros, J.; Jourdan, T.; Passilly-Degrace, P.; Fioramonti, X.; et al. Chemical Synthesis, Pharmacokinetic Properties and Biological Effects of JM-00266, a Putative Non-Brain Penetrant Cannabinoid Receptor 1 Inverse Agonist. *Int. J. Mol. Sci.* **2022**, *23*, 2923. https://doi.org/10.3390/ijms23062923

Academic Editor: Raffaele Capasso

Received: 26 January 2022
Accepted: 1 March 2022
Published: 8 March 2022

Publisher's Note: MDPI stays neutral with regard to jurisdictional claims in published maps and institutional affiliations.

Copyright: © 2022 by the authors. Submitted for possible open access publication under the terms and conditions of the Creative Commons Attribution (CC BY) license (https://creativecommons.org/licenses/by/4.0/).

Supplementary Materials

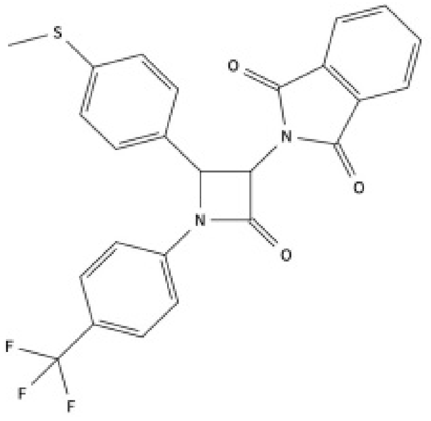

JM-00252

Figure S1. Structure of JM-00252.
